# Supplementary material for: A framework for dissecting affinities of multidrug efflux transporter AcrB to fluoroquinolones
Source: Commun Biol. 2022 Oct 6;5:1062. doi: 10.1038/s42003-022-04024-1 (PMC9537517; doi:10.1038/s42003-022-04024-1)
Supplement: Supplementary file 2 — Supplementary information [file 42003_2022_4024_MOESM2_ESM.pdf]

Supplementary Information

Supplementary Figure 1

<u>Fluoroquinolones (FQ)</u>

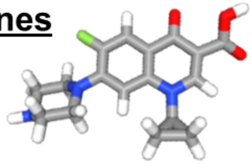
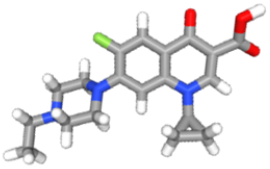
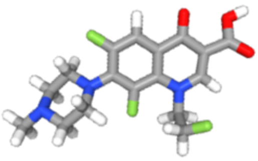
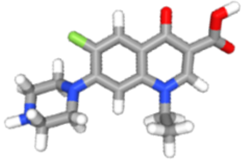

	Ciprofloxacin **CIP**	Enrofloxacin **ENR**	Fleroxacin **FLE**	Norfloxacin **NOR**
MW	331.34	359.39	369.34	319.34
Polar Surf. area	80.3	68.1	68.1	80.3
logD	-0.9	0.3	-0.1	-1.0
XLOGP3	-0.1	0.8	0.3	-0.3
Charge	0	0	0	0
Globularity	0.05	0.06	0.06	0.08
Rotatable bonds	3	4	4	3
$SICAR_{IN}$	29,727	29,477	16,320	28,990
$SICAR_{EFF}$	1.5	3.6	1.6	1.4

<u>Competitors (CPT)</u>

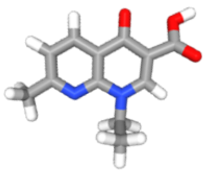
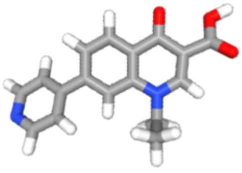
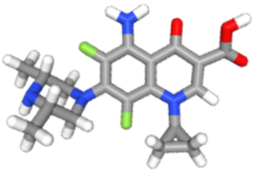

	Nalidixic acid **NAL**	Rosoxacin **ROS**	Sparfloxacin **SPA**
MW	231.23	293.30	392.41
Polar Surf. area	73.3	73.3	106.3
logD	-0.5	0.7	-0.1
XLOGP3	2.1	3.0	0.8
Charge	-1	-1	0
Globularity	0.05	0.03	0.06
Rotatable bonds	2	3	3

Supplementary Figure 1. Structures, physicochemical parameters, and accumulation indexes (SICAR) of the studied quinolones.

The four FQs [ciprofloxacin (CIP), enrofloxacin (ENR), fleroxacin (FLE) and norfloxacin (NOR)] have been selected according to (i) their ability to yield a strong fluorescent signal usable for fluorimetry analyses, (ii) their clinical and veterinary use, (iii) their structural similarities and, (iv) their previously reported different efflux

susceptibilities[1,2]. FQs exhibit the common quinolone structural core with an unsubstituted (CIP and NOR), methyl-substituted (FLE) or ethyl-substituted (ENR) R7 piperazine. ENR and CIP share an R1 cyclopropyl, NOR contains an R1 ethyl and FLE an R1 fluoro-ethyl. Only FLE has a substituent in R8, a fluorine. FQs have similar low molecular weight (MW) and a high polarity (ClogD < 1), are zwitterionic and are rather rigid molecules (number of rotatable bonds ≤ 4) with low globularity. CIP, NOR and ENR show similar high influx capacities ($SICAR_{IN}$) while FLE has a low influx index. CIP and NOR, FLE, ENR have low, medium and high efflux susceptibilities ($SICAR_{EFF}$), respectively[1].

Three quinolones (nalidixic acid (NAL), rosoxacin (ROS), and sparfloxacin (SPA)) with a negligible fluorescence signal under used conditions have been selected to compete with the FQs for the efflux system (competitors (CPTs)). NAL and ROS are first generation quinolones while SPA is a fourth generation FQ (with a R6-fluorine). NAL and ROS share an R1 methyl but have a R7 methyl (NAL) or pyridine (ROS). SPA shares with FQs an R1 cyclopropyl (ENR and CIP), an R7 piperazine, an R8 fluorine (FLE), but differs from FQs by an amino substituent in R5. SPA shares most of the physicochemical characteristics with the studied FQs apart from a relatively greater polar surface area. It is the most substituted and heavier compound even if its MW always remains < 400 Da. NAL and ROS are lighter compounds (MW < 300 Da). NAL exhibits only 2 rotatable bonds and ROS has the highest logD among the compounds studied even if it remains < 1 (0.7).

logD: logarithm of theoretical n-octanol/water partition coefficient corrected for ionization at pH 7.4, XLOGP3: logarithm of n-octanol/water partition coefficient for neutral forms, globularity: descriptor of molecular shape, rotatable bonds: number of flexible single bonds. $SICAR_{IN}$, $SICAR_{EFF}$: accumulation (SICAR) indexes from Ref[1].

Supplementary Figure 2

(µM)	EA27							EA294						
	SPA	ROS	NAL	CIP	ENR	FLE	NOR	SPA	ROS	NAL	CIP	ENR	FLE	NOR
0	100	100	100	100	100	100	100	100	100	100	100	100	100	100
1	100	100	100	96	95	99	100	100	100	100	97	90	100	100
2	100	97	100	91	92	99	100	100	96	100	91	76	100	100
4	100	100	100	88	91	98	100	96	95	98	64	41	100	96
8	100	100	100	84	86	99	100	65	87	99	28	16	96	98
16	100	99	100	61	74	99	100	40	66	100	17	7	63	89
32	100	100	100	28	44	89	96	28	36	100	11	8	31	66
64	89	94	100	14	18	58	87	17	18	94	10	4	19	22
128	33	87	100	9	11	28	58	10	6	74	7	2	17	11
256	11	76	96	4	7	14	18	4	3	44	4	0	13	2
512	5	76	85	0	2	12	4	3	3	20	2	0	7	0
1024	2	67	56	0	0	7	0	1	4	3	2	0	3	0

Metabolic activity (%)
- >90%
- 80-90%
- 70-80%
- 60-70%
- 50-60%
- 40-50%
- 30-40%
- 20-30%
- 10-20%
- <10%

Supplementary Figure 2. FQs and CPTs effects on metabolic activity of EA27 and EA294.

Effects of FQs and CPTs on EA27 and EA294 metabolic activities determined by the resazurin-reduction based assay. Actively metabolizing bacterial cells can reduce blue resazurin into red resorufin, which emits fluorescence. The resazurin reduction measured were translated into % metabolic activity and presented with color gradients (dark: 100% / light: 0%). Numbers indicate the corresponding % metabolic activity in EA27 and EA294 in presence of CPTs (SPA, ROS or NAL) and FQs (CIP, ENR, FLE, NOR).

Supplementary Figure 3

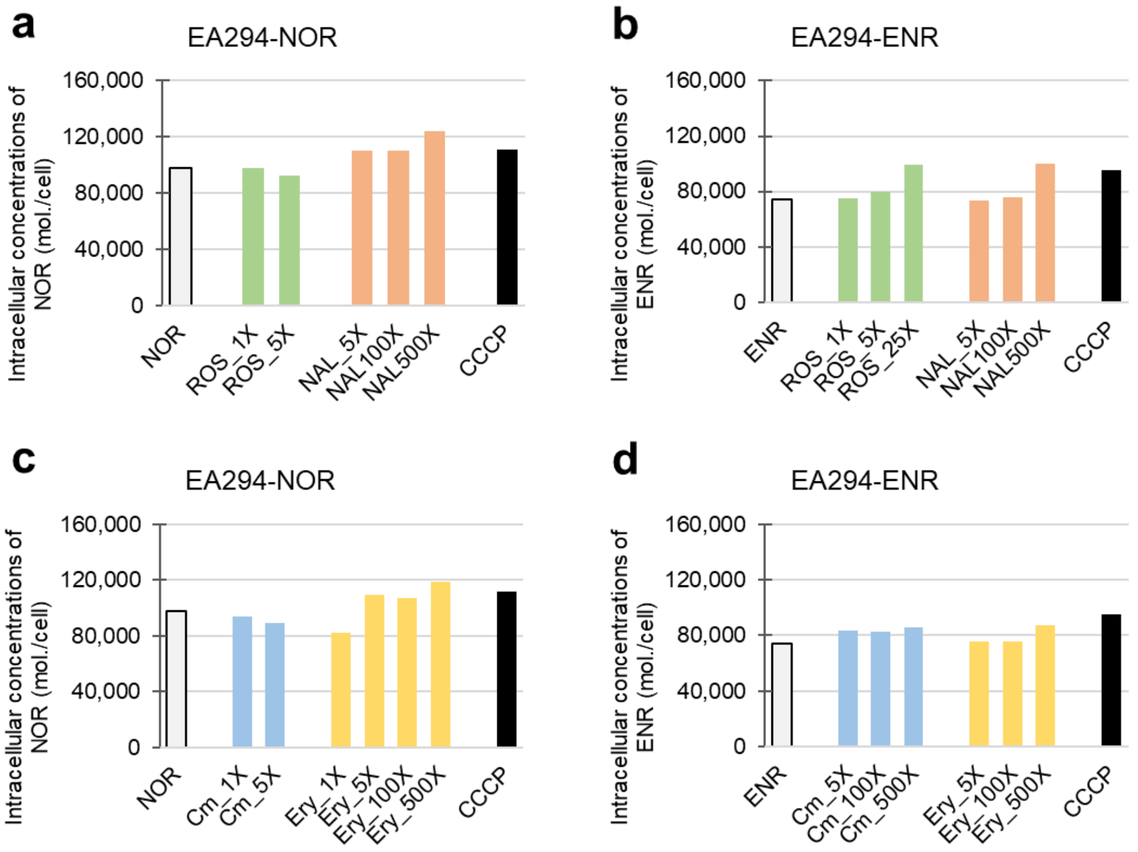

Supplementary Figure 3. ROS, NAL, Cm and Ery had no effect on the NOR and ENR accumulation in the no-efflux strain EA294.

NOR and ENR were co-incubated with ROS or NAL (**a**, **b**), Cm or Ery (**c, d**), in the no-efflux isogenic strain EA294 and compared to the accumulation measured in the overexpressing AcrB strain, EA27 **(Figure 1** and **Supplementary Figure 4**). The concentrations used during incubations were 8 µg/ml NOR/ENR, 20µM CCCP, X molar equivalent of ROS, NAL, Cm or Ery relative to the co-incubated NOR/ENR concentration. Bacterial suspensions were sampled at 20 minutes and intracellular concentrations of FQ were determined using spectrofluorimetry (see 'Methods').

Results were mean of triplicate obtained from one (**c, d**) or two (**a, b**) independent experiments.

Accumulation levels of both FQs were similar in EA294 whatever the co-incubated compound and whatever the presence of CCCP. These results validate the involvement of efflux pump saturation in the increased FQs accumulation observed in EA27 (**Figure 1** and **Supplementary Figure 4**).

Supplementary Figure 4

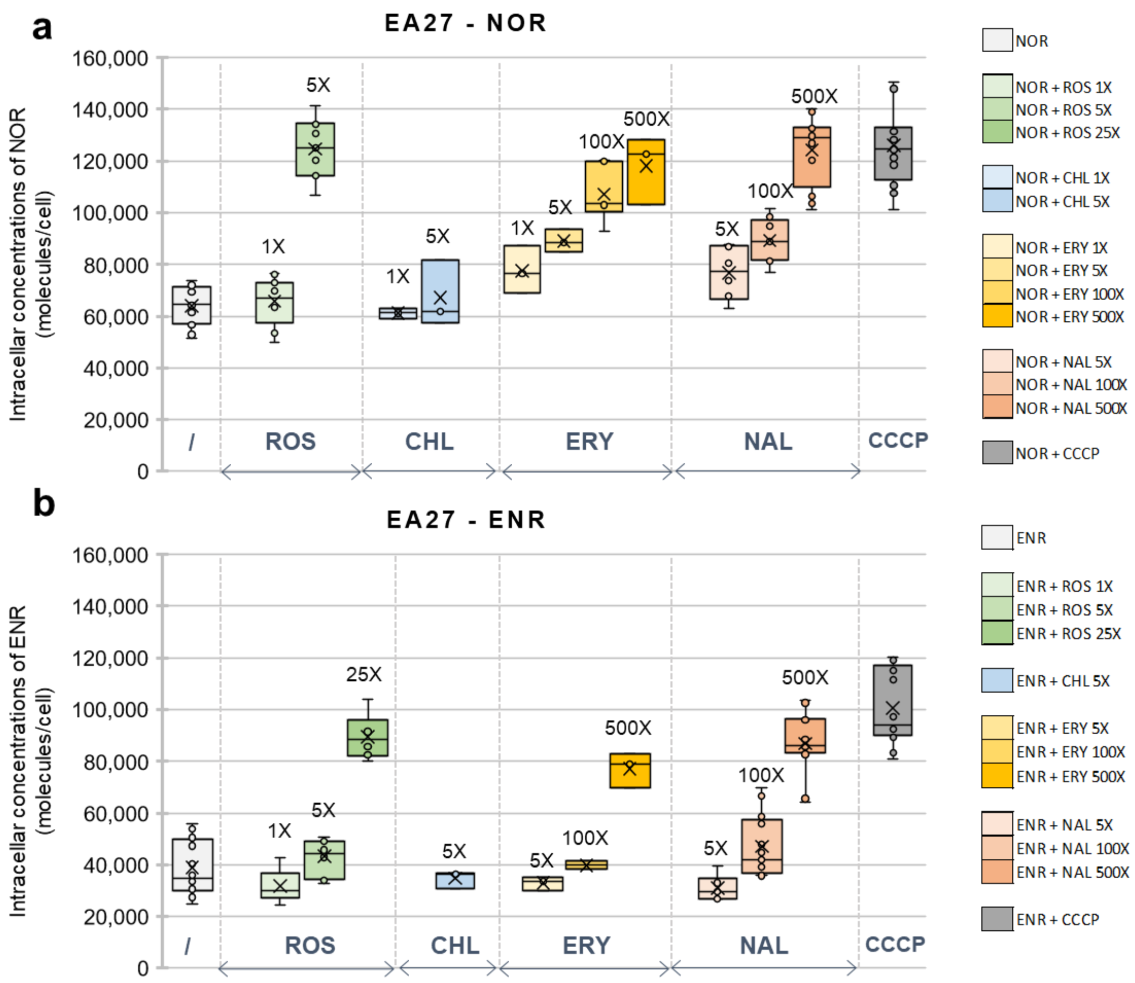

Supplementary Figure 4. Chloramphenicol and Erythromycin were as weak competitors as NAL.

Boxplot of NOR (**a**) and ENR (**b**) accumulation in EA27 incubated without or with CCCP, or with increasing concentrations of chloramphenicol (CHL) or erythromycin (ERY). Results from same experiments performed with the CPTs ROS and NAL were plotted for comparison. The concentrations were 8 µg/ml NOR/ENR, 20µM CCCP, X molar equivalent of chloramphenicol/erythromycin relative to the co-incubated NOR/ENR concentration. Chloramphenicol 100X and 500X having induced a non negligeable cell killing, the corresponding data was not shown. Bacterial suspensions were sampled at 20 minutes and intracellular concentrations of NOR/ENR were

determined using spectrofluorimetry (see ‘Methods’). n=3, 3, 6, 7 for competition with chloramphenicol, erythromycin, ROS and NAL, respectively.

Supplementary Figure 5

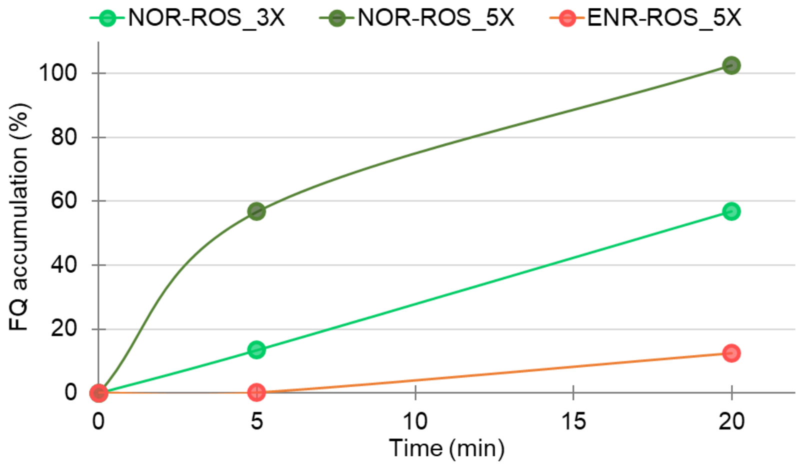

Supplementary Figure 5. ROS effect on NOR accumulation is time dependent.

To investigate the effect of CPT co-administration on the early stages of FQ accumulation, data were extracted at a shorter incubation time of 5 minutes. ENR or NOR at 8 µg/ml were co-incubated in EA27 with ROS 3X or 5X (relative to ENR/NOR concentration in molar equivalent) during 5 and 20 minutes. Results are means obtained from two independent experiments performed in triplicate.

Accumulation of NOR co-incubated with ROS for 5 and 20 minutes amounted to 13% and 57% respectively with ROS 3X and to 57% and 103% with ROS 5X. Thus, ROS was able to compete with NOR already after 5 minutes. On the other hand, no notable difference in the ENR accumulation was observed after 5 or 20 minutes of co-incubation with ROS 5X. The steady-state of competition reached more rapidly for NOR-ROS than for ENR-ROS could reflect a higher affinity to AcrB for ENR than for ROS or NOR.

Supplementary Figure 6

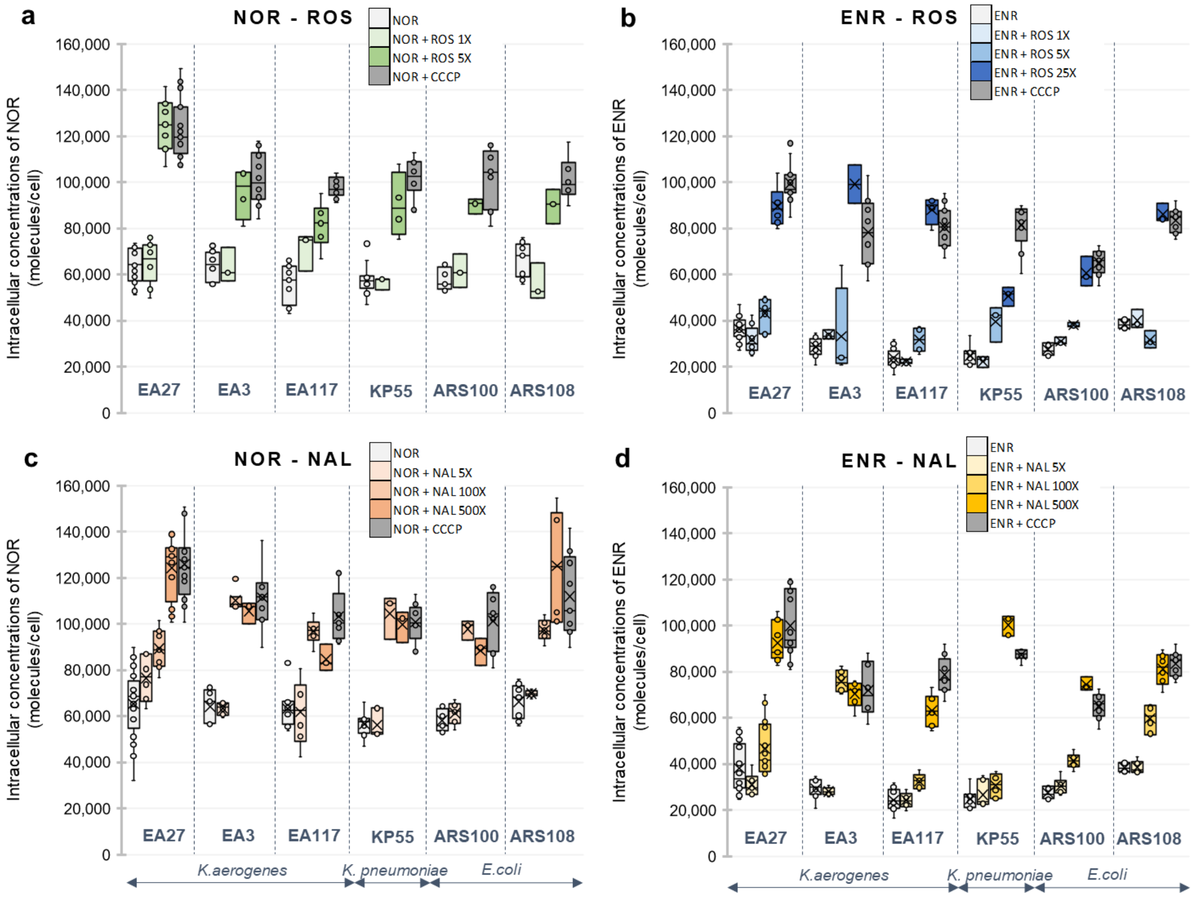

Supplementary Figure 6. NOR-ROS was the best pair for efflux pump saturation in various strains and species of MDR clinical isolates.

Boxplot of ENR (**a**, **c**) and NOR (**b**, **d**) accumulation incubated without or with CCCP, or with increasing concentrations of ROS (**a**, **b**) or NAL (**c**, **d**) in various MDR clinical strains of *K. aerogenes* (EA27, EA3, EA117), *K. pneumoniae* (KP55), *E. coli* (ARS100, ARS108). The concentrations were 8 µg/ml ENR/NOR, 20µM CCCP, X molar equivalent of ROS/NAL relative to the co-incubated ENR/NOR concentration. Bacterial suspensions were sampled at 20 minutes and intracellular concentrations of NOR/ENR were determined using spectrofluorimetry (see 'Methods').

Supplementary Figure 7

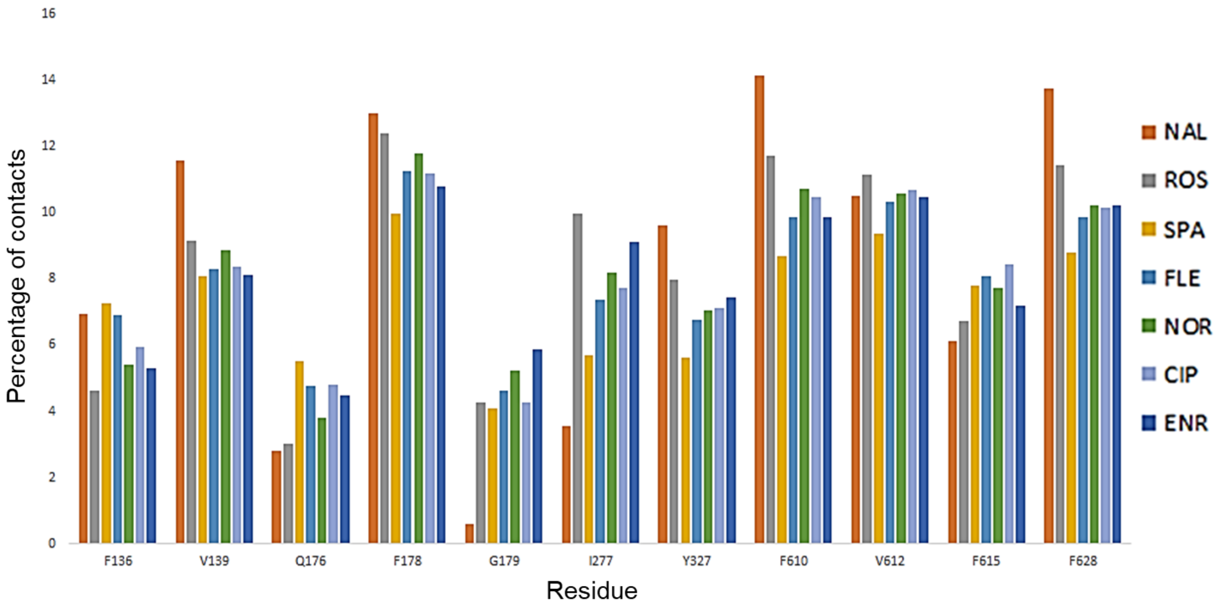

Supplementary Figure 7. Statistical contact analysis of docking poses. Percentage of contacts of each ligand with residues of the DP_T of AcrB. Only protein residues with frequencies higher than 5% with at least one ligand are reported.

Supplementary Figure 8

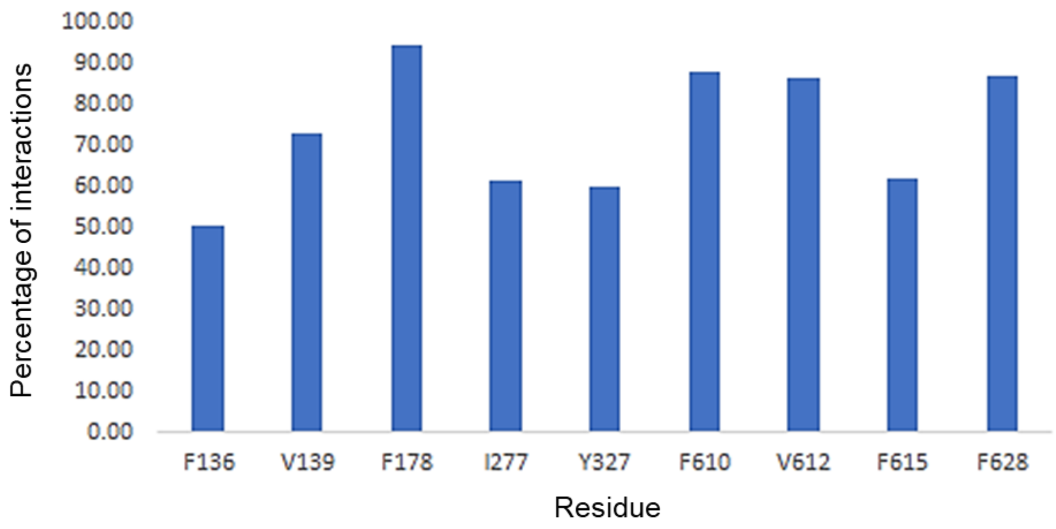

Supplementary Figure 8. Percentage of contacts per residue considering all docking poses generated for all compounds.

Only protein residues that are in contact with more than 50% of all docking poses are reported.

Supplementary Figure 9

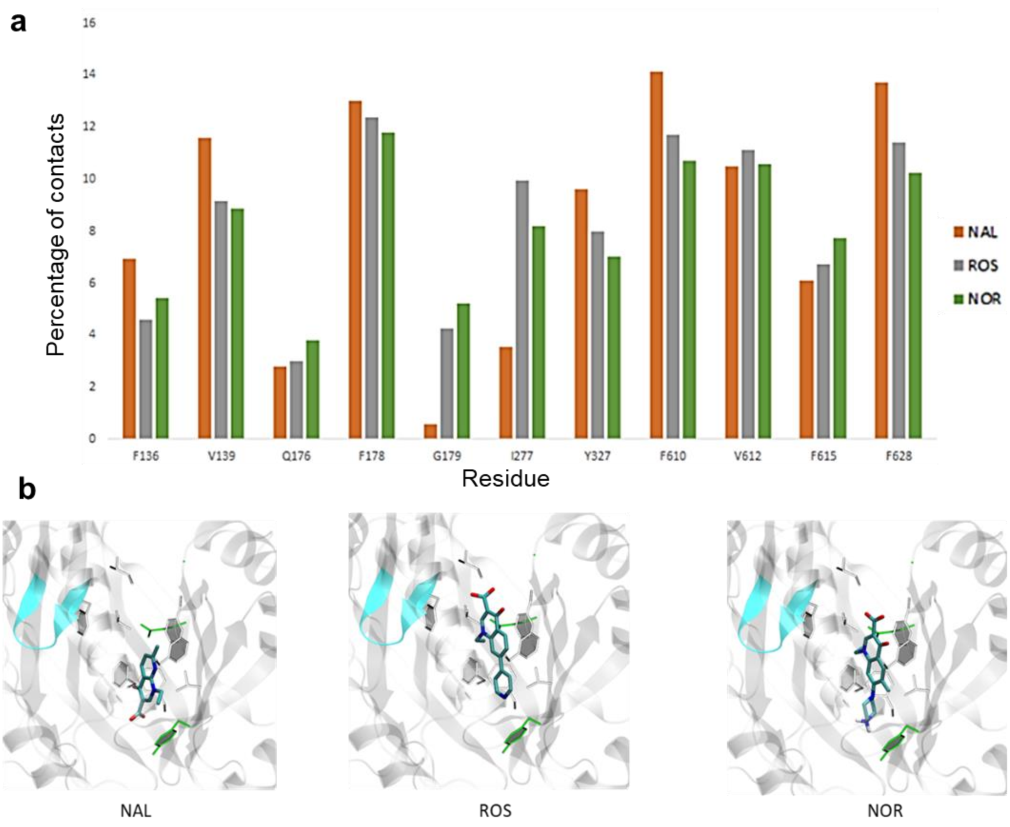

Supplementary Figure 9. a Same as Supplementary Figure 7 only for NAL, ROS and NOR. **b** Best docking poses of NAL, ROS and NOR in the DP_T of AcrB. Protein residues with more than 5% of contacts are highlighted (see Figure 5). The switch loop is shown as cyan solid tube.

Supplementary Figure 10

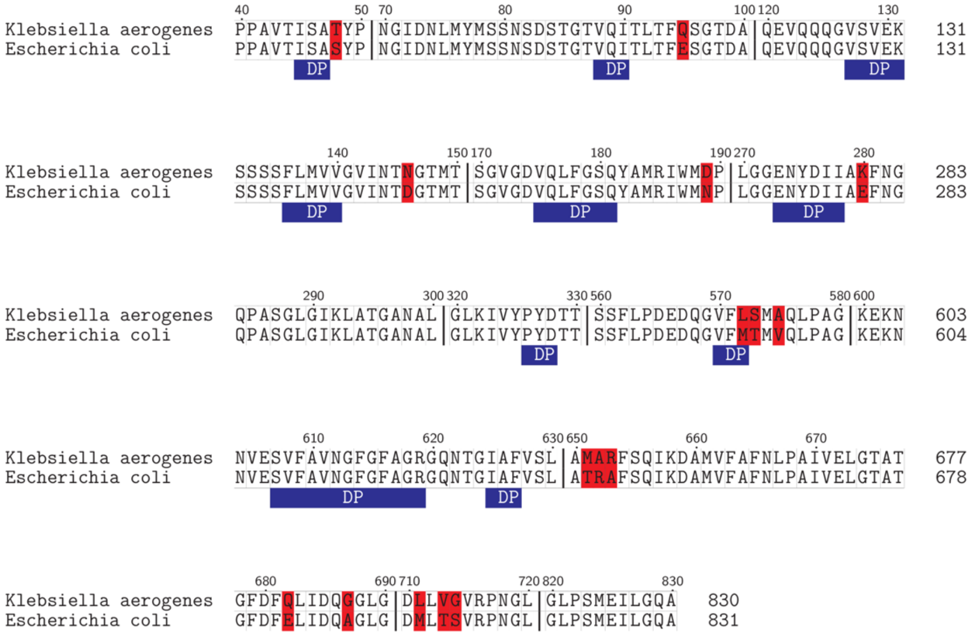

Supplementary Figure 10. Alignment of protein sequences of the computationally relevant regions of *Klebsiella aerogenes* and *Escherichia coli*. The differences are highlighted in red, while the residues that compose the deep binding pocket (DP) are indicated by a blue bar. Only residue M572 is part of both groups. The alignment was represented with TeXshade[3], and the Uniprot accession code numbers of the two sequencies are Q9AEG1 for *Klebsiella aerogenes*, and P31224 for *Escherichia coli*, respectively.

Supplementary Note 1. Statistical contact analysis of docking poses.

The predicted binding affinity for SPA is comparable to that of FQs, consistently with the susceptibilities assays experiments reported in this study. The large overlap among the binding poses of all compounds (**Figure 5**) can be evaluated more quantitatively by comparing the percentage of contacts established by each compound with the DP_T residues (**Supplementary Figure 7**).

All compounds show similar contact frequencies well above a threshold of 5% with residues V139, F178, F610, V612, F615 and F628, confirming the tendency of all molecules to sit in the same preferred location within the pocket. Q176 and G179, the only two non-hydrophobic residues, appear to be prevalent for only one compound, respectively SPA and ENR, with contact frequencies slightly above 5%. Interestingly, NAL has very few contacts with G179 and differs from the other compounds for the frequency of contacts to I277. This latter residue is characterized by a well pronounced peak for ROS.

To further assess the importance of each residue for the whole set of compounds we collected in **Supplementary Figure 8** the percentage of contacts by considering all docking poses; only residues with percentage higher than 50% are shown. Overall, out of 24 AAs contacted at least by one ligand at least once, 15 of them have contacts in less than half of the generated poses (S46, Q89, S128, E130, S134, Q176, L177, G179, S180, E273, N274, D276, M573, F617, R620, not reported in **Supplementary Figure 8**), 5 are contacted between 50% and 80% (F136, V139, I277, Y327, F615) and only 4 are found in 80% or more of the poses (F178, F610, V612, F628). The most contacted AAs are hydrophobic (F136, F178, Y327, F610, F615, F628) and only 3 out of 9 are aliphatic (V139, I277, V612). On the other hand, of the other 15 AA only 3 are

hydrophobic (L177, M573 and F617), while the rest are polar (apart from G179). These results agree with previous studies on AcrB[4].

Focusing on the NAL, NOR and ROS triad, the interaction patterns show similarities between NOR and ROS, which establish a similar percentage of contacts with all the 11 important residues (**Supplementary Figure 9a**). On the other hand, NAL behaves in a noticeable different way in 7 out of 11 cases (F136, V139, G179, I277, Y327, F610, F628) where the difference between the contact percentage is at least ±1.5%.

In particular, although being the smallest compound, NAL contacts more frequently a series of residues (V139, F178, Y327, F610, F628) than the other ligands (**Supplementary Figure 7**). NAL also contacts F136 more than NOR and ROS. On the other hand, NAL is the only compound having very few contacts with G179 and I277, mainly due to its reduced size as compared to the others. This is evident from **Supplementary Figure 9b** where the best poses of NAL and NOR are shown for comparison. The presence of an extra aliphatic ring in NOR, absent in NAL, allows for contacts between the molecule and G179 and I277. Additionally, G179 does not have a side chain able to establish a stabilizing interaction with the scaffold of the ligands. Q176 is also characterized by a reduced number of contacts with the three quinolones; this is due to the polar nature of the residue, which hampers a stabilizing contribution to the compound-pocket interaction. On the other hand, F136, V139, F610 and F628 exhibit a higher percentage of contacts with NAL than the other 2 molecules. Again, this is likely due to the smaller size of NAL which allows for a sampling of a larger set of configurations within the pocket.

Supplementary References

1. Vergalli, J. *et al.* The challenge of intracellular antibiotic accumulation, a function of fluoroquinolone influx versus bacterial efflux. *Communications Biology* **3**, 1–12 (2020).
2. Vergalli, J. *et al.* Fluoroquinolone structure and translocation flux across bacterial membrane. *Sci. Rep.* **7**, 9821 (2017).
3. Beitz, E. TeXshade: shading and labeling of multiple sequence alignments using LaTeX2e. *Bioinformatics* **16**, 135–139 (2000).
4. Ruggerone, P., Vargiu, A. V., Collu, F., Fischer, N. & Kandt, C. Molecular dynamics computer simulations of multidrug RND efflux pumps. *Comput Struct Biotechnol J* **5**, (2013).
