# Supplementary material for: A framework for dissecting affinities of multidrug efflux transporter AcrB to fluoroquinolones
Source: Commun Biol. 2022 Oct 6;5:1062. doi: 10.1038/s42003-022-04024-1 (PMC9537517; doi:10.1038/s42003-022-04024-1)
Supplement: Supplementary file 5 — Reporting Summary-New [file 42003_2022_4024_MOESM5_ESM.pdf]

nature portfolio

Corresponding author(s): Jean-Marie PAGES

Last updated by author(s): Sep 7, 2022

Reporting Summary

Nature Portfolio wishes to improve the reproducibility of the work that we publish. This form provides structure for consistency and transparency in reporting. For further information on Nature Portfolio policies, see our Editorial Policies and the Editorial Policy Checklist.

Statistics

For all statistical analyses, confirm that the following items are present in the figure legend, table legend, main text, or Methods section.

n/a	Confirmed	
☐	☒	The exact sample size (n) for each experimental group/condition, given as a discrete number and unit of measurement
☐	☒	A statement on whether measurements were taken from distinct samples or whether the same sample was measured repeatedly
☐	☒	The statistical test(s) used AND whether they are one- or two-sided<br>*Only common tests should be described solely by name; describe more complex techniques in the Methods section.*
☐	☒	A description of all covariates tested
☐	☒	A description of any assumptions or corrections, such as tests of normality and adjustment for multiple comparisons
☐	☒	A full description of the statistical parameters including central tendency (e.g. means) or other basic estimates (e.g. regression coefficient) AND variation (e.g. standard deviation) or associated estimates of uncertainty (e.g. confidence intervals)
☐	☒	For null hypothesis testing, the test statistic (e.g. F, t, r) with confidence intervals, effect sizes, degrees of freedom and P value noted<br>*Give P values as exact values whenever suitable.*
☒	☐	For Bayesian analysis, information on the choice of priors and Markov chain Monte Carlo settings
☒	☐	For hierarchical and complex designs, identification of the appropriate level for tests and full reporting of outcomes
☒	☐	Estimates of effect sizes (e.g. Cohen's d, Pearson's r), indicating how they were calculated

Our web collection on statistics for biologists contains articles on many of the points above.

Software and code

Policy information about availability of computer code

Data collection	Na
Data analysis	The microscopy images were analyzed with a set of python scripts in a jupyter notebook still in active development, and not yet ready for broader use (https://gitlab.synchrotron-soleil.fr/disco-beamline/bacteria-drug-uv-analysis)

For manuscripts utilizing custom algorithms or software that are central to the research but not yet described in published literature, software must be made available to editors and reviewers. We strongly encourage code deposition in a community repository (e.g. GitHub). See the Nature Portfolio guidelines for submitting code & software for further information.

Data

Policy information about availability of data

All manuscripts must include a data availability statement. This statement should provide the following information, where applicable:

- Accession codes, unique identifiers, or web links for publicly available datasets
- A description of any restrictions on data availability
- For clinical datasets or third party data, please ensure that the statement adheres to our policy

Source data are available (Supplementary Data 1). All other data supporting this study are available within the article and its Supplementary Information or are available from the corresponding author on reasonable request.

Field-specific reporting

Please select the one below that is the best fit for your research. If you are not sure, read the appropriate sections before making your selection.

☒ Life sciences ☐ Behavioural & social sciences ☐ Ecological, evolutionary & environmental sciences

For a reference copy of the document with all sections, see nature.com/documents/nr-reporting-summary-flat.pdf

Life sciences study design

All studies must disclose on these points even when the disclosure is negative.

Sample size	Accumulation and killing assays (Figures 1 and 4) : n ≥ 3.<br>Data of Figure 2 were calculated from data of Figure 1.<br>Microscopy data (Figure 3) : n = 312, 423, 200, 288, 336, 205, 321, 274 bacterial cells incubated with NOR, NOR+CCCP, NOR+ROS 3X, NOR +ROS 5X, NOR+SPA 5X, NOR+SPA 50X, NOR+NAL 50X, NOR+NAL 500X.<br>Figure 6 : Average binding affinity (docking score) and standard deviations from 180 binding modes (n=180)<br>Table 1 : Three independent assays (n = 3).<br>Table 3 : Average binding affinity and corresponding standard deviation for the most populated conformational cluster (n about 400).<br>Table 4: Binding free energies shown in Table 4 were from 3 independent MD simulations.
Data exclusions	Fluorescence signals from cells overlapping or close to the edges of the microscope field were excluded from the microscopy data (Figure 3). Technical replicates from accumulation assays were excluded when their fluorescence signals were outside the linear part of the calibration curves.
Replication	Biological assays corresponding to Figures 1, 2, 4, Table 1 and supplementary figure 4 were carried out in triplicate and at least three independent assays were performed.<br>Microscopy experiments were repeated at two independent times, each with 6 distinct microscopy fields containing about 20 distinct cells (figure 3).<br>One or two independent assays were performed in triplicate to obtain the data from Supplementary Figures 3, 5, 6.
Randomization	Not relevant in this study
Blinding	Not relevant in this study

Reporting for specific materials, systems and methods

We require information from authors about some types of materials, experimental systems and methods used in many studies. Here, indicate whether each material, system or method listed is relevant to your study. If you are not sure if a list item applies to your research, read the appropriate section before selecting a response.

Materials & experimental systems

n/a	Involved in the study	
☒	☐	Antibodies
☒	☐	Eukaryotic cell lines
☒	☐	Palaeontology and archaeology
☒	☐	Animals and other organisms
☒	☐	Human research participants
☒	☐	Clinical data
☒	☐	Dual use research of concern

Methods

n/a	Involved in the study	
☒	☐	ChIP-seq
☒	☐	Flow cytometry
☒	☐	MRI-based neuroimaging
